# Treatment of secondary hip arthritis from shell fragment and gunshot injury in the Syrian civil war

**DOI:** 10.1186/s13018-020-01993-z

**Published:** 2020-10-08

**Authors:** Raif Özden, Serkan Davut, Yunus Doğramacı, Aydıner Kalacı, İbrahim Gökhan Duman, Vedat Uruç

**Affiliations:** Department of Orthopaedics and Traumatology, Faculty of Medicine, Mustafa Kemal University, Serinyol, 31001 Antakya, Hatay Turkey

**Keywords:** Arthroplasty, Hip gunshot, Secondary hip osteoarthritis, Shell fragment injuries

## Abstract

**Background:**

In gunshot and shell fragment injuries to the hip joint, orthopedic intervention includes wound assessment and care, osteosynthesis of fractures, and avoiding of infection and osteoarthritis. Individuals injured in the Syrian civil war were frequently transferred to the authors’ institution in neighboring city. Orthopedic trauma exposures were determined in approximately 30% of these patients. The aim of this study was to evaluate the outcomes of the patients with secondary hip arthritis due to prior gunshot and shell fragment (shrapnel) injuries who underwent primary total hip arthroplasty.

**Methods:**

This retrospective study reviewed 26 patients (24 males, 2 females) who underwent hip arthroplasty due to prior gunshot and shell fragment injuries from November 2013 to January 2019. For all patients, the Harris Hip Score (HHS) was evaluated preoperatively and after surgery.

**Results:**

Mean age was 31.5 (range, 19–48) years. The mean preoperative HHS was 52.95 points, and the mean postoperative HHS was 79.92 points at the final follow-up after surgery. Patients with shell fragment injuries to the hip joint had higher infection rates, but it is not statistically significant.

**Conclusions:**

An anatomic reduction of the fracture may not be possible in these cases as a result of significant bone and/or cartilage loss. Total hip arthroplasty can be done after gunshot- and shell fragment-related posttraumatic arthritis. It is an effective treatment choice to reduce pain and improve function, but the surgeon must be very careful because of high rate of infection.

## Background

Gunshot wounds to the hip joint constitute 2% of all extremity and 4% of lower extremity injuries. If nonarticular wounds to the hip area are included, these percentages increase to 9% and 17%, respectively [1]. Due to the social and medical problems associated with civilian war, millions of refugees had to immigrate to the neighboring countries [2]. Until March 2016, it has been estimated that the total number of recorded and unrecorded refugees reached 2.7 million in our country [3]. Individuals injured in the Syrian civil war were frequently transferred to the authors’ institution in neighboring city. Most of the cases admitted to general surgery department, division of neurosurgery, and department of orthopedics of the same hospital [4, 5]. Orthopedic trauma exposures were determined in approximately 30% of these patients [6]. Between 2011 and 2019, 10,006 cases were treated in the department of orthopedics. Of these, 276 adult patients had hip joint injury.

Shell fragments are different shapes and sizes of high-energy fragments and cause extensive tissue destruction. They are different from bullets that produce high-energy transfer to the tissue by creating a temporary cavitation [7]. While the number of gunshot and shell fragment wounds increases, orthopedic surgeons are experienced with more penetrating musculoskeletal injuries. Orthopedic intervention includes wound assessment and care, osteosynthesis of fractures, and avoiding of infection and osteoarthritis. Other considerations include penetration of abdominal organs, retained intraarticular fragments, spinal canal involvement, and vascular injury. In the non-symptomatic patient, bullets and shell fragments can be left in place. In the bone and soft tissue, the retained fragments are surrounded by fibrotic avascular scar tissue preventing lead dissolution and migration [8]. However, the literature has documented some cases of systemic lead intoxication in patients whose bullets are in contact with synovial and cerebrospinal fluid [9]. A bullet that passes into the hip joint rarely causes an infection, but a transabdominal trajectory that subsequently enters the hip joint indicates a high risk for infection after total hip arthroplasty [10].

If the function of the hip joint cannot be reestablished, the effective solutions are either arthrodesis or total joint replacement. The purpose of this study was to evaluate the outcomes and complications associated with total hip arthroplasty in 26 patients with secondary arthritis due to prior gunshot and shell fragment injuries.

## Materials and methods

**Fig. 1:**
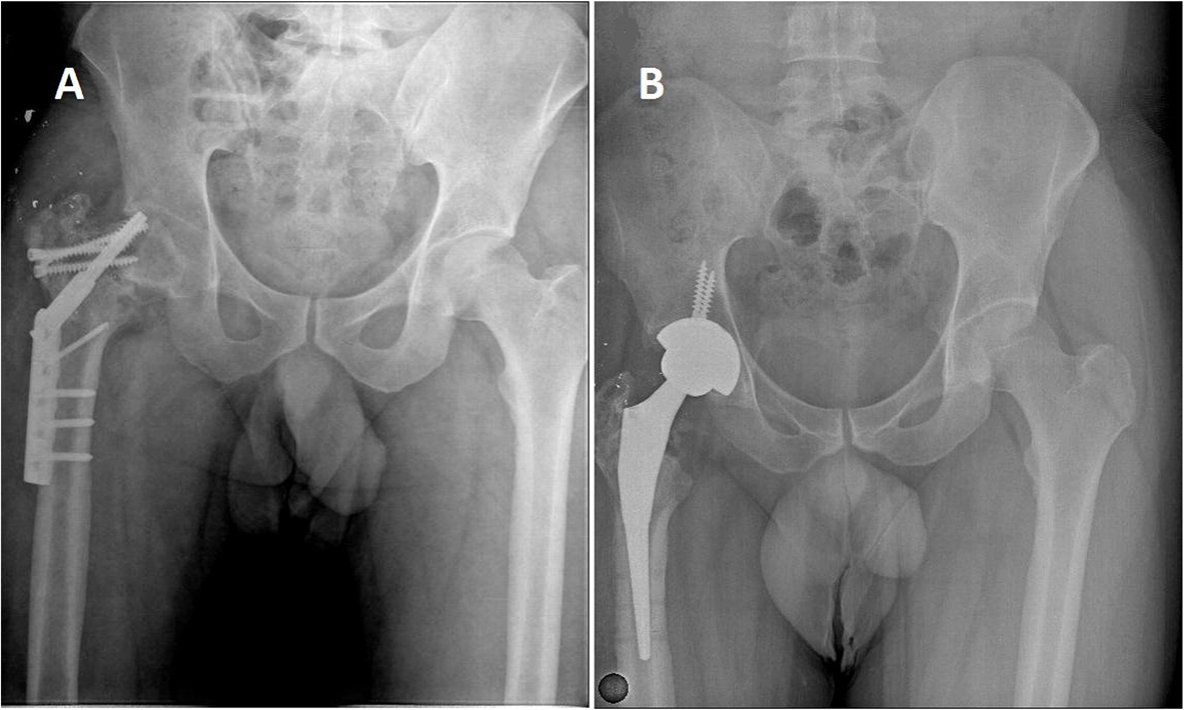
A 33-year-old male patient with right intertrochanteric fracture and classified as group 2 (shell fragment). **a** Dynamic hip screw was used for the treatment of fracture. **b** After 15 months, total hip arthroplasty was performed due to pseudoarthrosis and hip osteoarthritis

This retrospective study was approved by the local ethics committee of the hospital. Informed consent was obtained from all patients before the start of study. Twenty-six patients treated in a single center between November 2013 and January 2019 were reviewed (24 males, 2 females). Patients were divided into 2 groups: group 1, patients with gunshot (bullet) injury (14 patients), and group 2, patients injured with shell fragment (12 patients). Exclusion criteria were extensive soft tissue injury around the hip, total neurologic deficit, patients with incomplete medical records or who were lost during follow-up, and open fractures associated with arterial injury requiring vascular repair. Patient demographics are shown in Table 1. Previous interventions for joint injury before total hip arthroplasty (Fig. 1) and preoperative and postoperative Harris Hip Score (HHS) are shown in Table 2. Acute orthopedic treatment included wound evaluation and care, tetanus prophylaxis, and a minimum of 72 h of prophylactic intravenous antibiotics. Gentamycin, metronidazole, and cefazolin were used in all cases in acute phase of injury.

**Table 1:** Patient demographics

Parameter	Value
Number of patients	26
Number of hips	26
Age (years)	31.5 (19–48)
Male patients	24 (92.3%)
Female patients	2 (7.6%)
Velocity	
High velocity	14 (53.8%)
Shell fragment	12 (46.1%)
Side	
Right	12 (46.1%)
Left	14 (53.8%)
Revision total hip prosthesis	5 (19.23%)
Dislocation of prosthesis	1 (3.84%)
Infection (bullet)	2 (14.3%)
Infection (shell fragment)	4 (33.3%)
Total infection rate	6 (23%)
The average time from injury to arthroplasty	15.7 months (6–48 months)
Mean follow-up	47.2 months (12–85 months)

**Table 2:** Previous interventions of hip joint injury before total hip arthroplasty and HHSs of patients before and after surgery

Patient	Age	Group	Preoperative surgery	Skeletal surgery	Preoperative HHS	Postoperative HHS
1	21	1	External fixation	Pertrochanteric	41.95	82
2	35	1	Debridement	Femoral neck	63.95	91.5
3	19	2	Debridement	Acetabulum	52.7	84.8
4	35	2	Internal fixation	Pertrochanteric	52.45	75.5
5	35	1	Debridement	Acetabulum, femoral head	29.25	73.7
6	33	2	Internal fixation	Femoral neck	51.25	79.8
7	31	2	Debridement	Femoral head and neck	51.7	63.9
8	42	2	Internal fixation	Femoral neck	62.95	94
9	34	2	Debridement	Femoral head and neck	65.95	70
10	27	1	External fixation	Pertrochanteric, femoral head	71.95	72.5
11	24	1	Debridement	Acetabulum, femoral head	45.95	79
12	27	1	Debridement	Femoral neck	58.95	75
13	42	2	Debridement	Acetabulum, femoral head	46.6	83.7
14	21	2	Debridement	Acetabulum	64.2	94
15	32	2	Debridement	Acetabulum, femoral neck	49.7	81.3
16	42	2	Debridement	Acetabulum	60.7	84
17	29	1	Debridement	Acetabulum, femoral neck	39.65	77.8
18	28	1	Debridement	Acetabulum, femoral head	41.95	75.8
19	24	2	Debridement	Femoral head and neck	31.1	46.8
20	27	1	Debridement	Femoral head and neck	52.7	88.7
21	41	1	Debridement	Femoral neck	50.9	82.6
22	24	1	Debridement	Acetabulum	50.9	92.6
23	31	1	Internal fixation	Acetabulum	44.5	84.8
24	39	1	Debridement	Femoral neck	56.7	89
25	48	1	Internal fixation	Pertrochanteric, femoral neck	72.95	85.85
26	29	2	Debridement	Acetabulum	60.95	69.5

### Statistical analysis

All statistical analysis was performed with IBM SPSS Statistics for Windows, version 21.0 (IBM Corp., USA). The preoperative and postoperative HHS, age, and sex distribution were tested for statistical significance between the two groups with the Mann-Whitney *U* test. Wilcoxon’s test was used both to compare the difference of preoperative HHS and postoperative HHS among all patients (26 patients) and to compare the preoperative and postoperative HHS of the two groups among themselves. Infection rates between the two groups and infection associated with intraarticular fragment were tested with chi-square test or Fisher’s exact test. A *P* value of less than 0.05 (*P* < 0.05) was considered statistically significant.

### Surgical management

**Fig. 2:**
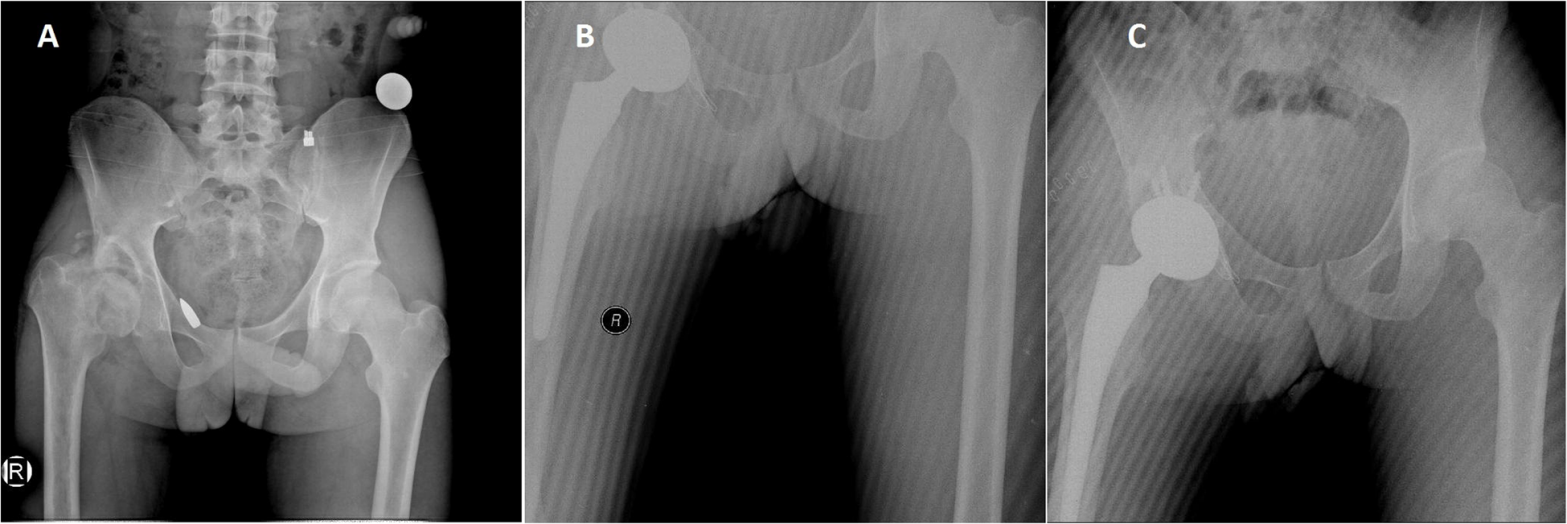
A 35-year-old male patient. Patient was classified as group 1 (bullet). **a** Right femoral neck fracture. **b** Total hip arthroplasty was performed and bullet was removed

**Fig. 3:**
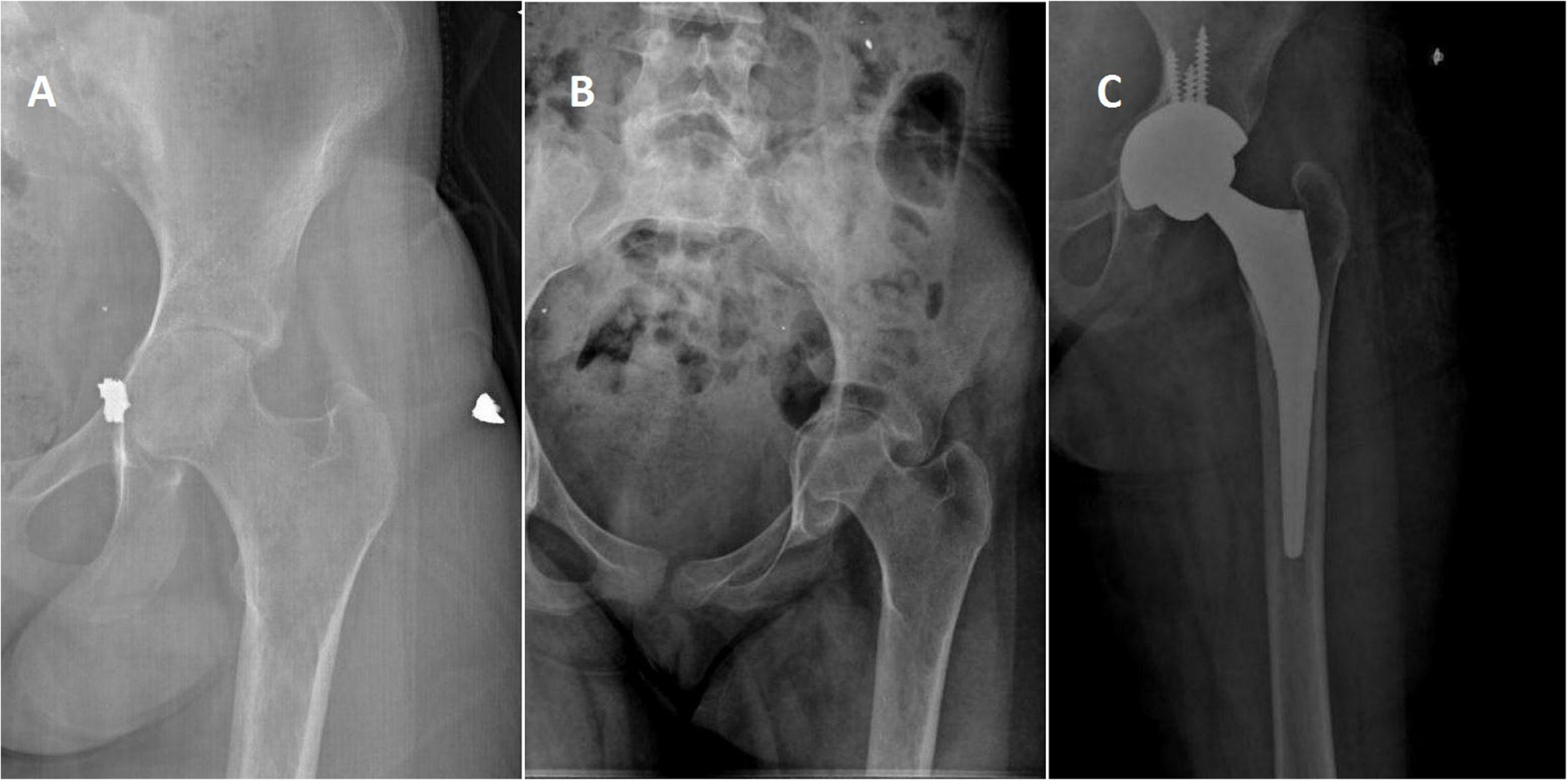
A 19-year-old female patient. Left acetabular fracture and classified as group 2 (shell fragment). **a** Anteroposterior radiograph of the pelvis. **b** Shell fragments were removed. **c** Nine months later, arthroplasty was performed

**Fig. 4:**
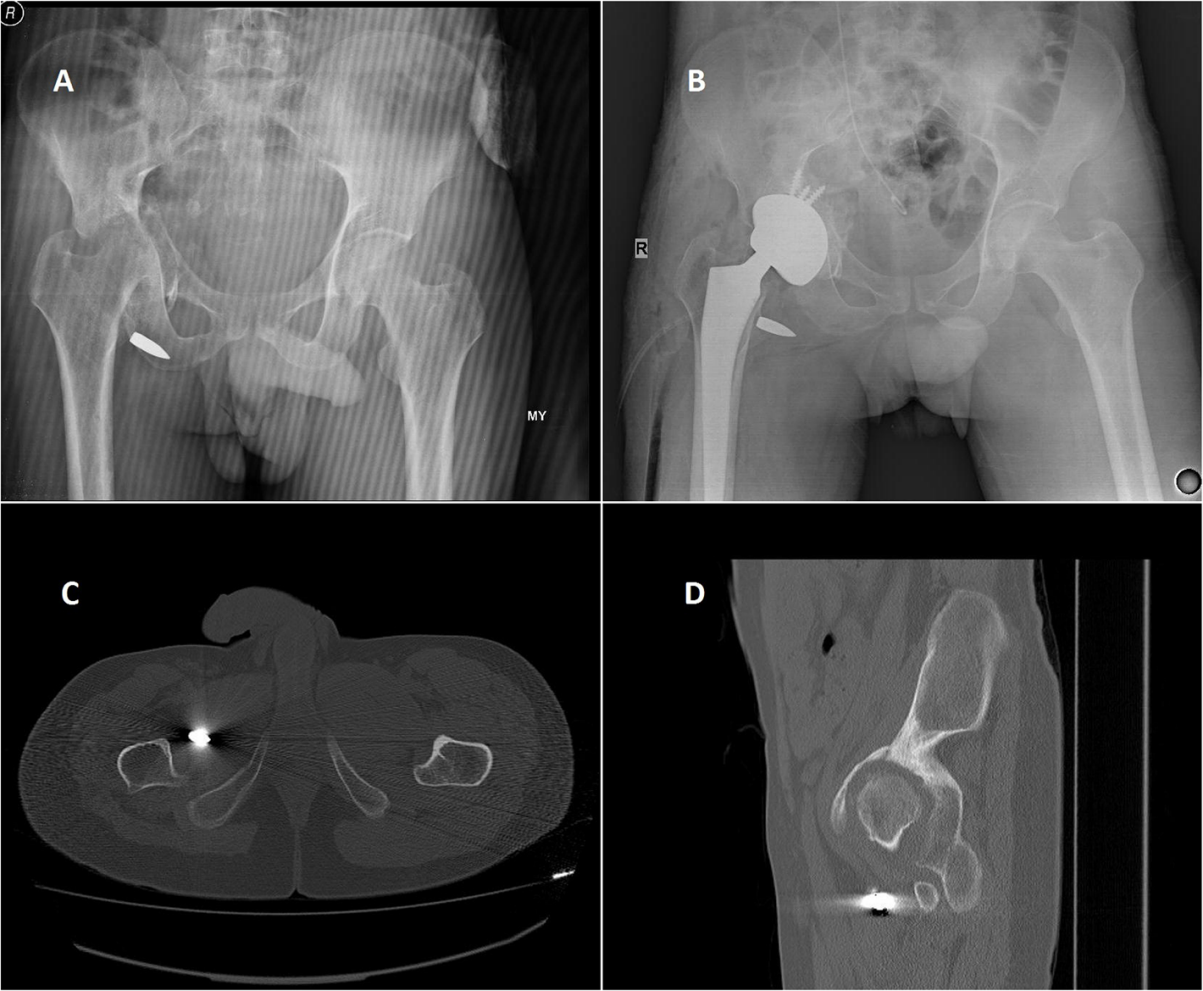
A 35-year-old male patient. Right acetabular fracture. No internal fixation was performed and classified as group 1 (bullet). **a** Six months after initial injury, there was severe hip osteoarthritis. **b** After 13 months, total hip arthroplasty was performed. **c**, **d** Computerized axial and sagittal tomography scan of the hip, showing the bullet around the trochanter minor. Bullet was not removed because of difficulty in finding the bullet and other risks of surgical intervention

**Fig. 5:**
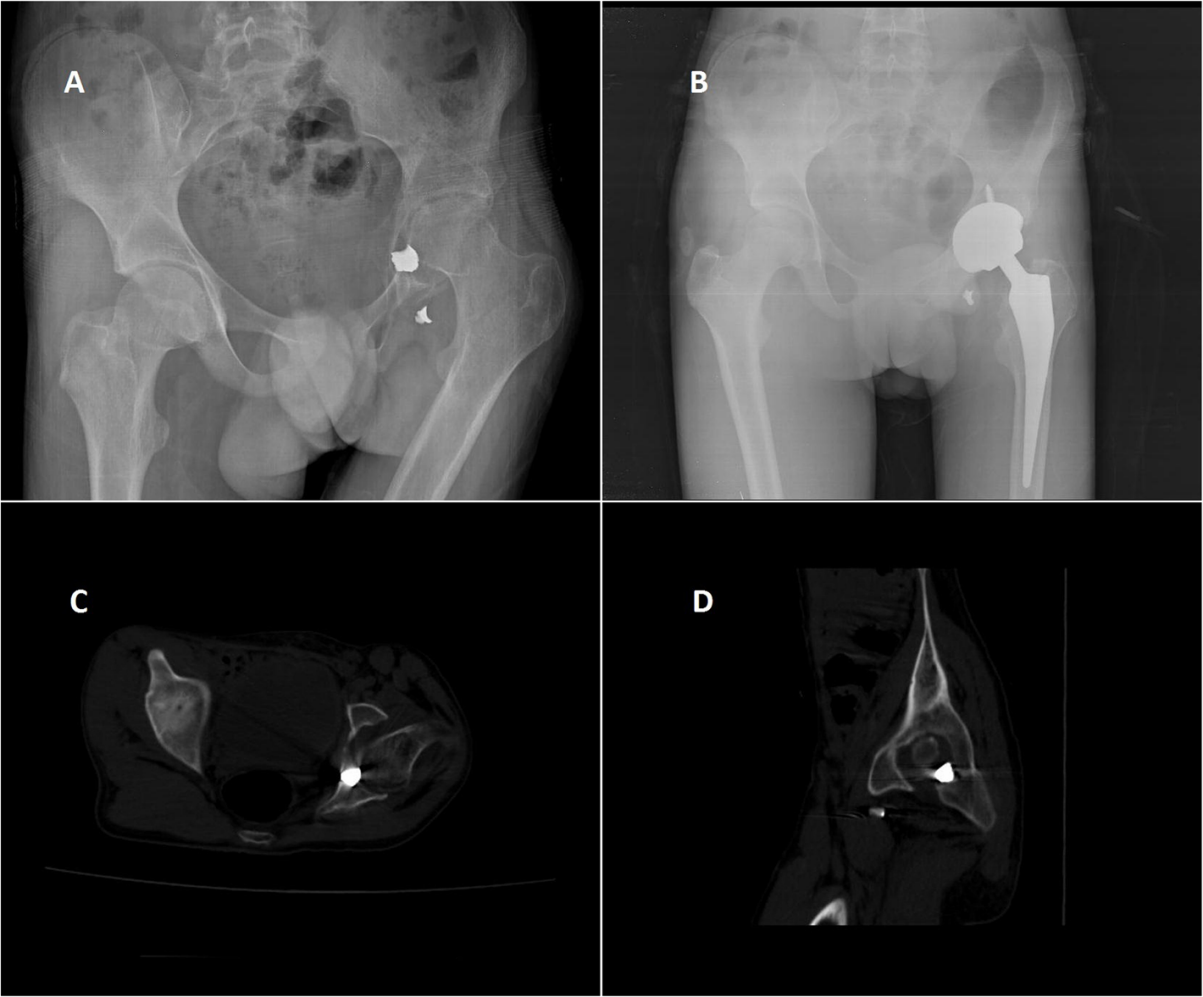
A 20-year-old male patient. **a** Left acetabular fracture and classified as group 2 (shell fragment). **b** After 8 months, total hip arthroplasty was performed. **c**, **d** Computerized axial and sagittal tomography scan of the hip, showing shell fragment in the hip joint

Hip arthroplasty was performed for patients who developed osteoarthritis due to gunshot or shell fragment injury and with no sign of infection (Figs. 2 and 3). All patients were treated by two orthopedic senior surgeons via posterolateral approach. Cementless femoral and acetabular components were used for the total joint arthroplasty (Zimmer Biomet, Warsaw, IN, USA). Cefazolin was used for prophylaxis and continued postoperatively until the drain was removed about 48 h. Low molecular weight heparin was administered for 4 weeks. Patients were allowed for partial weight-bearing within the first 24 h after surgery. After 4 weeks, patients were permitted for full weight-bearing. The patients were discharged about 4–6 days postoperatively if there was no wound problem. When there was prolonged wound drainage, discharge was delayed to about 7–10 days. Pelvis anteroposterior view was taken immediately after surgery, 45 days, and 3, 6, and 12 months at each follow-up examination. Nonroutine radiographic analysis was performed as a result of a change in the clinical condition and laboratory tests of the patient. In patients with intraarticular or even extraarticular bullet fragments that could not be removed, we took care for lead intoxication (Figs. 4 and 5). Patients were assessed according to functional outcome using HHS [11], prosthesis infection, and infection related with intraarticular fragment.

## Results

**Fig. 6:**
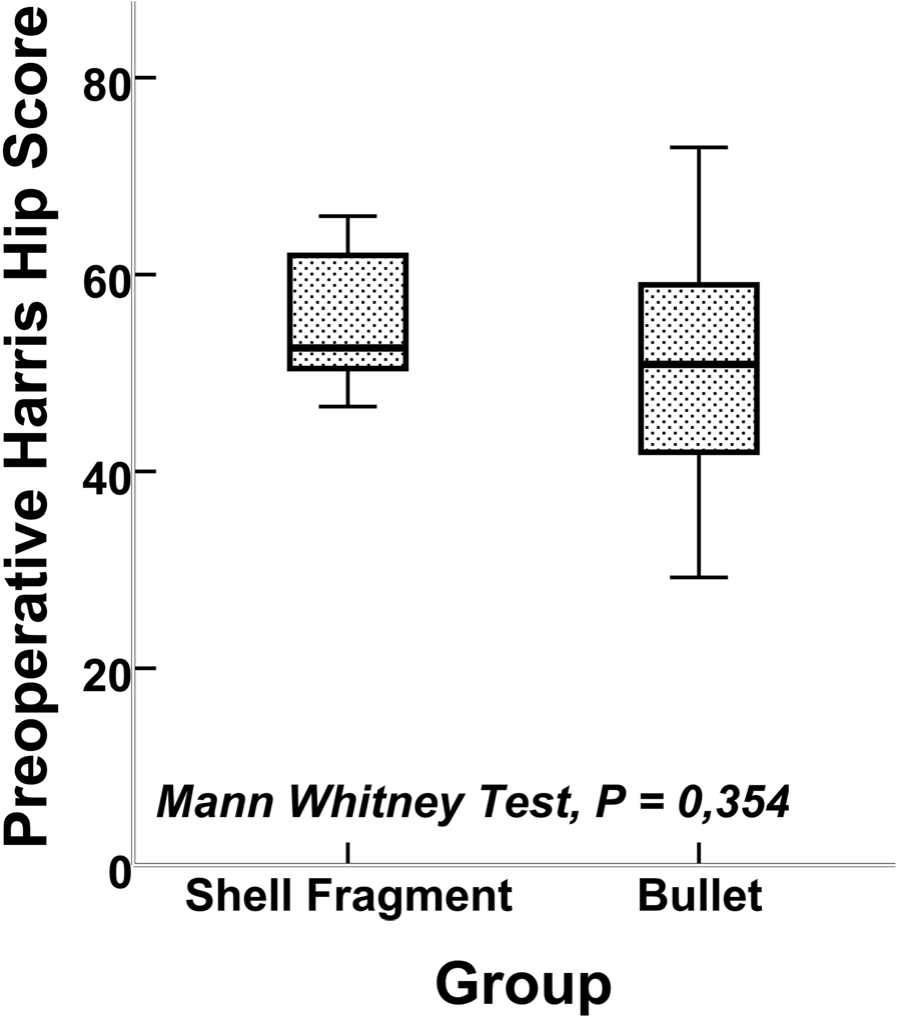
Preoperative mean HHSs of the groups

**Fig. 7:**
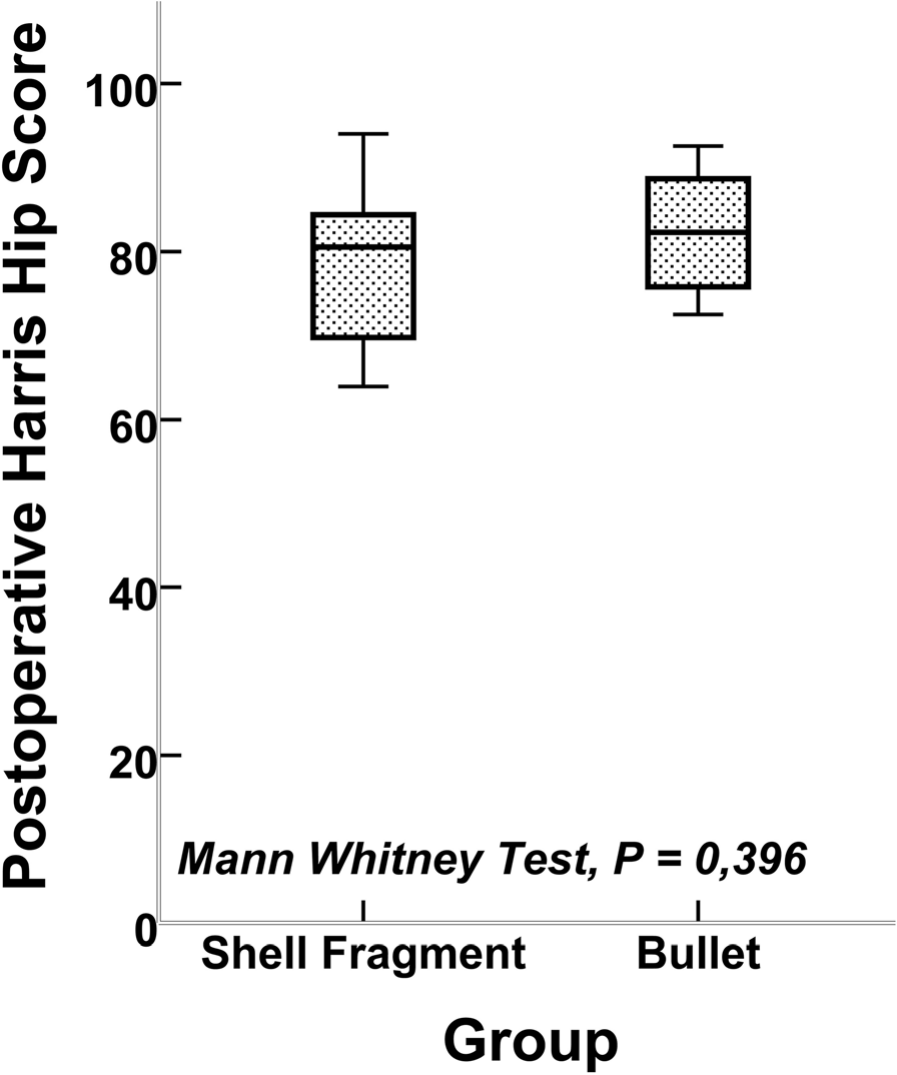
Postoperative mean HHSs of the groups

The average time from initial injury to total hip arthroplasty was 15.7 months (range, 6–48 months). Patients were those with a minimum of 1 year postsurgery with a mean follow-up of 47.2 months (range, 12–85 months). The mean age of the patients at the time of surgery was 31.5 years (range, 19–48 years). The mean preoperative HHS was 52.95 points (range, 29.25–72.95 points) and 79.92 points (range, 46.8–94 points) after total hip arthroplasty among all patients at the final follow-up. The mean preoperative and postoperative HHS in group 1 was 51.91 points (range, 29.25–72.95 points) and 82.20 points (range, 72.5–92.6 points), respectively (Fig. 6). The mean preoperative HHS was 54.18 points (range, 31.10–65.95 points), and postoperative HHS was 77.27 points (range, 46.8–94 points) in group 2 (Fig. 7). Mean preoperative and postoperative HHS is shown in Table 3. No evidence of lead toxicity was detected in any of the patients during follow-up. Two of the 14 patients (14.3%) injured with bullet and 4 of the 12 patients (33.3%) with shell fragment injury had postoperative infection. The total infection rate was 23% (in 26 patients). Resection arthroplasty was done to one patient in group 2. In the other five patients, prosthesis was removed and antibiotic-loaded cement spacer was placed, and when there was no sign of infection, revision total hip prosthesis was performed. In one patient in whom dislocation occurred after arthroplasty, closed reduction was performed successfully.

**Table 3:** Mean preoperative and postoperative HHS of patients and *P* values

Group	Mean preoperative HHS (min–max)	Mean postoperative HHS (min–max)	*P* value
Group 1	51.91 (29.25–72.95)	82.20 (72.50–92.60)	*P* = 0.001*
Group 2	54.10 (31.1–65.95)	77.20 (46.80–94)	*P* = 0.002*
Group 1 + group 2	52.96 (29.25–72.95)	79.92 (46.80–94)	*P* < 0.001*

*Wilcoxon’s test

There was no statistically significant difference according to age, sex, preoperative HHS (*P* = 0.354), and postoperative HHS (*P* = 0.396) between the two groups. There was a statistically significant difference between preoperative HHS and postoperative HHS in total group (*P* < 0.001). Also, the two groups were compared among themselves and postoperative HHS was significantly different from preoperative HHS in group 1 (*P* = 0.001) and group 2 (*P* = 0,002) (Table 3). Infection rates between the two groups were not statistically different (*P* = 0.365). There was 1 patient with intraarticular fragment in group 1, and there were 5 patients in group 2. These fragments were removed at initial surgery. None of the patients was infected, so intraarticular fragments were not associated with high risk of infection after total hip arthroplasty (*P* = 0.280).

## Discussion

To our knowledge, there is a paucity of analogous studies which have been done on the hip. Although the classification, treatment, and complications of hip fractures in blunt trauma are well known, there is limited information on gunshot and shrapnel injuries. An anatomic reduction of the fracture may not be possible in these cases as a result of significant bone and/or cartilage loss. In these cases, primary stabilization of the fracture site is attempted rather than an anatomic reconstruction of the joint to prevent further complications such as secondary displacement or pseudarthrosis.

Firstly, an anteroposterior radiograph of the pelvis is needed to analyze bone and joint involvement, and pelvic instability [12]. Gunshot and shell fragment wounds to the pelvis require special attention because of the vital anatomy with in the pelvis. Bladder, urinary tract, and bowel injuries must be aggressively investigated. At the time of presentation, injury to vital structures may lead to associated morbidity and mortality; hence, these patients should be treated in a multispecialty center [12]. After a treatment plan has been made for life-threatening conditions, a detailed orthopedic evaluation is essential. Most of the combat injuries are penetrative, and most are caused by fragments from explosive munitions rather than by bullets fired by military arms [13]. In shrapnel injuries, there may be multiple small fragments which are varying in size and can be easily overlooked. Twelve patients evaluated in the current study were injured with shell fragment.

In the acute period, arthrodesis or arthroplasty is not recommended. After hip joint injury, open reduction and internal fixation are advised for fracture treatment [6]. Arthroplasty should be done under elective conditions because of the risk of infection. In the present study, hip arthroplasty was performed in the chronic period after hip joint gunshot and shell fragment injuries as in previous studies [10, 14, 15].

In patients with posttraumatic arthritis which occurred after gunshot wounds, surgery is more difficult than primary total hip arthroplasty [14]. An initial open reduction for acetabular fracture may compromise the outcome of a subsequent total hip arthroplasty by disturbing the blood supply of the acetabulum and by initiating the development of heterotopic bone, dense scar tissue, and ischemic muscle or bone. In addition, anatomical change or defect of the acetabulum and previous fixation devices may be a problem [16, 17].

A wound that contains splits in the skin but has small skin loss can be closed without more extensive skin grafting or flap coverage. The majority of patients with low velocity gunshot wounds, uncontaminated injuries of skin, and fractures not requiring operative stabilization can be safely treated nonoperatively with superficial irrigation and cleaning of wound followed by a dressing, with or without antibiotics and outpatient treatment. In more extensive wounds with skin loss, first treatment should be managed in the operating room. Longitudinal incisions of the skin and fascia should be done to reduce pressure, remove the debris and hematoma, and expose the underlying muscle. Surgical removal of skin infrequently is indicated for the initial surgery, other than removing irregular margins of the entrance and exit wounds [18, 19]. Contaminated high-velocity gunshot injuries are indications for more aggressive and multiple surgical debridements and administration of prophylactic antibiotics [20]. Negative pressure dressings can be used for the primary management of more extensive wounds to reduce the size of the defect needing coverage [21].

Relatively minimal skeletal muscle necrosis occurred if the blood circulation is intact. Excision has been suggested for skeletal muscle that would not survive. For wounds in which there is a simple perforation of the limb, there is a small rim of devitalized cell. The wound tract will heal without any problem if permitted to drain. For wounds in which there is more extensive skeletal muscle damage, a comprehensive exploration of the wound is warranted [19].

Compartment syndrome has been shown related with gunshot wounds [22, 23]. The amount of swelling in a compartment after a gunshot wound may range from minimal to involvement of the whole compartment. Involvement of the entire compartment is uncommon but can arise when patients have extensive soft tissue and vascular injury or injury causing ischemia. Patients with a big hematoma, vascular injury, or extensive swelling at first evaluation are candidates for more aggressive surgical procedure [19].

Bullets do not become sterile after friction and heating in the barrel. Patzakis et al. [24] divided 310 cases with open fractures into three groups. Seventy-eight caused by gunshot wounds. Four of 78 wounds became infected; 1 of them was osteomyelitis. The authors assigned the infection to severity of injury in 3 of the patients who had shotgun wounds with severe soft tissue injury. A fourth infection was in the no-antibiotic group. Neviaser and Clawson [25] reported that initial focus on the abdominal injury with neglect of associated acetabular fractures can lead to delayed diagnosis of the hip involvement, which finally leads to septic arthritis and joint destruction. If an infected nonunion or malunion was present with resorption of the femoral head, an antibiotic spacer should be needed before total hip arthroplasty.

Heterotopic ossification can occur after pelvic and hip trauma. The prevalence of heterotopic ossification in war wounded patients is higher than in civilian trauma. A substantial portion of wartime soft tissue trauma occurs at the time of injury, and additional soft tissue trauma in the form of multiple surgical debridements as well as muscle dissection during internal fixation of the fracture may result in a higher degree of muscle damage and may cause an increased frequency of heterotopic ossification [26]. Conversion to total hip arthroplasty may be more difficult.

If the abductor muscle length is not preserved due to fracture of the femoral neck and displacement of the trochanter, femoral shortening is necessary [10]. Shortening of the femur is important to be able to reduce the risk of damaging neurovascular structures due to extreme limb lengthening. Also, reduction of the femoral head into the true acetabulum remains demanding without shortening the femur [27].

Transabdominal gunshot wounds of the pelvis and hip are those that traverse the abdomen before entering the pelvis or hip. The orthopedic treatment of patients with transabdominal injuries remains more controversial. For transabdominal gunshot injuries including the hip joint, algorithms were reported suggesting immediate irrigation and debridement with antibiotic treatment [28]. In study patients treated with arthroplasty following a gunshot injury to the hip joint, those contaminated with intestinal flora had worst Harris Hip Score. Very high infection rates were seen in patients with accompanying intestinal injury [10]. Bullet may be a source for infection and thereby warrant operative debridement and bullet removal [29, 30]. Conversely, it was reported that gunshot wounds to the pelvis and spine with associated abdominal organ trauma do not warrant operative debridement of fractures and bullet paths, or bullet removal. Parenteral antibiotics are sufficient treatment [31–33]. In addition, it is reported that the rate of infection is directly related to the treatment of the abdominal injury and not to the debridement of the tract, fracture, or bullet removal [34, 35]. Transpelvic bullets that traverse the urinary bladder require urologic management to avoid infection or fistula formation. When an associated injury to the femoral artery is suspected, an arteriogram is indicated. If a vascular injury is present, an emergent repair or bypass is necessary. Mortality is more common when vascular injuries were combined with rectal injuries [36]. In the current study, there was only one patient with intestinal injury and infection did not occur after arthroplasty.

Rhee and Martin [37] advised that removal of foreign body is indicated when symptomatic in soft tissue and all penetrated hips be explored to remove possible foreign bodies. Long et al. [38] recommended arthrotomy for entire patients with high-energy injuries and hip penetration, intraarticular fractures entailing internal fixation, and retained articular bullets or fragments; however, in patients with low-energy wounds with hip penetration without articular bullets or fragments and without requiring internal fixation, arthrotomy is not necessary.

At our institution, the first step was always wound irrigation and debridement to minimize the risk of infection. In the current study, all patients with gunshot wounds had high-velocity injuries. Triple prophylactic antibiotics were administered routinely for all gunshot and shell fragment injuries. If it was possible, retained fragment was removed. In our study, in 22 cases, surgical debridement was performed once while the other 4 patients needed multiple debridements. There are reports of arthroscopic removal of fragments from the joints [39, 40]. The minimally invasive approach may decrease the morbidity associated with muscle and soft tissue dissection; however, it marginally increased operative times and the possibility of compartment syndrome resulting from leakage of irrigation fluid, through capsular and fascial defects into the muscular compartments. Authors have no experience with this technique.

The mechanism of joint destruction is the motion of irregular joint surfaces that may be formed after the initial injury, joint sepsis, lead arthropathy, foreign body reaction, synovitis, the presence of intraarticular small pieces of bone and cartilage, and metallic fragments into the joint. Shell fragments can do the same things as bullets. Exposure of a bullet to synovial fluid may lead to dissolution of the leaded fragments due to the presence of hyaluronic acid and the low pH of synovial fluid. Thus, patients with intraarticular fragments require removal because they may lead to mechanical abrasion and joint destruction [20, 37, 41–44].

Naziri et al. [14] compared the patients who underwent primary total hip arthroplasty for degenerative joint disease and secondary arthritis due to prior gunshot wound injuries and found similar results in the two groups. In another study, 4 patients who developed posttraumatic arthritis from gunshot injuries to their hips underwent successful total hip arthroplasty without complications [15]. Unlike previous studies, in the current study, infection rates were high and about 23% of the cases, although in our study there is no control group. When compared with literature, infection rates were high according to primary total hip arthroplasty. It was stated that infection rates after primary total hip replacement surgery vary from 0.4 to 1.4%, with most infections occurring during the first year [45–48].

There are limitations of the present study. First, the study design was retrospective without a control group; second, the number of patients was small; there were more patients about 51 cases, but these patients had incomplete medical records or were lost during follow-up. If all of the patients were enrolled in the study, the result could be different.

In this small series of patients, the authors have shown that failed attempts to treat these patients operatively or nonoperatively can be addressed with total hip arthroplasty to reduce pain and improve function and range of motion at mid-term follow-up. It is an effective treatment choice to reduce pain and improve function, but the surgeon must be very careful because of high rate of infection. Further studies with longer follow-up are needed.

## Data Availability

The datasets generated and analyzed during the current study are available from the corresponding author on reasonable request.

## Abbreviations

HHS: Harris Hip Score

## References

[1] Dougherty PJ, Vaidya R, Silverton CD, Bartlett C, Najibi S (2009). Joint and long-bone gunshot injuries. J Bone Joint Surg Am.

[2] Çelikel A, Karaarslan B, Demirkıran DS, Zeren C, Arslan MM (2014). A series of civilian fatalities during the war in Syria. Ulus Travma Acil Cerrahi Derg.

[3] AFAD (2016) Republic of Turkey Prime Ministry. Disaster and Emergency Management Presidency. Syrian refugees in Turkey, 2013: field survey results. https://www.afad.gov.tr/Dokuman/TR/61-2013123015505-syrian-refugees-in-turkey-2013-print12.11.2013-eng.pdf. Accessed 16 June 2016.

[4] Akkucuk S, Aydogan A, Yetim I, Ugur M, Oruc C, Kilic E, Paltaci I, Kaplan A, Temiz M (2016). Surgical outcomes of a civil war in a neighbouring country. JR Army Med Corps.

[5] Aras M, Altaş M, Yilmaz A, Serarslan Y, Yilmaz N, Yengil E, Urfali B (2014). Being a neighbor to Syria: a retrospective analysis of patients brought to our clinic for cranial gunshot wounds in the Syrian civil war. Clin Neurol Neurosurg.

[6] Karakus A, Yengil E, Akkucuk S, Cevik C, Zeren C, Uruc V (2013). The reflection of the Syrian civil war on the emergency department and assessment of hospital costs. Ulus Travma Acil Cerrahi Derg.

[7] Shuker ST (2016). Emergency management of high-energy shell fragment midface complex injuries. J Craniofac Surg.

[8] Apte A, Bradford K, Dente C, Smith RN (2019). Lead toxicity from retained bullet fragments: a systematic review and meta-analysis. J Trauma Acute Care Surg.

[9] Dienstknecht T, Horst K, Sellei RM, Berner A, Nerlich M, Hardcastle TC (2012). Indications for bullet removal: overviewof the literature, and clinical practice. Guidelines for European trauma surgeons. Eur J Trauma Emerg Surg.

[10] Pazarci O, Kilinc S, Camurcu Y, Bulut O (2019). Total hip arthroplasty after hip joint gunshot injury. J Orthop Surg (Hong Kong).

[11] Söderman P, Malchau H (2001). Is the Harris Hip Score system useful to study the outcome of total hip replacement?. Clin Orthop Relat Res.

[12] Zura RD, Bosse MJ (2003). Current treatment of gunshot wounds to the hip and pelvis. Clin Orthop Relat Res.

[13] Champion HR, Bellamy RF, Roberts CP, Leppaniemi A (2003) A profile of combat injury. J Trauma54:13-19. doi.10.1097/01.TA.0000057151.02906.27. 10.1097/01.TA.0000057151.02906.27 12768096

[14] Naziri Q, Abraham R, Scollan JP, Mixa PJ, Cherkalin D, Varghese JJ, Newman JM, Maheshwari AV (2017). Primary total hip arthroplasty for gunshot injury-induced secondary arthritis of the hip: what are the outcomes?. J Hip Surg.

[15] Naziri Q, Issa K, Rizkala A, Rasquinha VJ, Pivec R, Harwin SF, Mont MA (2013). Posttraumatic arthritis from gunshot injuries to the hip requiring a primary THA. Orthopedics.

[16] Mears DC, Velyvis JH (2001). Primary total hip arthroplasty after acetabular fracture. Instr Course Lect.

[17] Sierra RJ, Mabry TM, Sems SA, Berry DJ (2013). Acetabular fractures: the role of total hip replacement. Bone Joint J.

[18] Bartlett CS (2003). Clinical update: gunshot wound ballistics. Clin Orthop Relat Res.

[19] Dougherty PJ, Najibi S, Silverton C, Vaidya R (2009). Gunshot wounds: epidemiology, wound ballistics, and soft-tissue treatment. Instr Course Lect.

[20] Bartlett CS, Helfet DL, Hausman MR, Strauss E (2000). Ballistics and gunshot wounds: effects on musculoskeletal tissues. J Am Acad Orthop Surg.

[21] Leininger BE, Rasmussen TE, Smith DL, Jenkins DH, Coppola C (2006). Experience with wound VAC and delayed primary closure of contaminated soft tissue injuries in Iraq. J Trauma.

[22] Moed BR, Fakhouri AJ (1991). Compartment syndrome after low-velocity gunshot wounds to the forearm. J Orthop Trauma.

[23] Foster RD, Albright JA (1990). Acute compartment syndrome of the thigh: case report. J Trauma.

[24] Patzakis MJ, Harvey JP, Ivler D (1974). The role of antibiotics in the management of open fractures. J Bone Joint Surg Am.

[25] Neviaser RJ, Clawson RS (1976). Transabdominal gunshot wounds of the hip. South Med J.

[26] Forsberg JA, Pepek JM, Wagner S, Wilson K, Flint J, Andersen RC, Tadaki D, Gage FA, Stojadinovic A, Elster EA (2009). Heterotopic ossification in high-energy war-time extremity injuries: prevalance and risk factors. J Bone Joint Surg Am.

[27] Neumann D, Thaler C, Dorn U (2012). Femoral shortening and cementless arthroplasty in Crowe type 4 congenital dislocation of the hip. Int Orthop.

[28] Najibi S, Matta JM, Dougherty PJ, Tannast M (2012). Gunshot wounds to the acetabulum. J Orthop Trauma.

[29] Poret HA, Fabian TC, Croce MA, Bynoe RP, Kudsk KA (1991). Analysis of septic morbidity following gunshot wounds to the colon: the missile is an adjuvant for abscess. J Trauma.

[30] Romanick PC, Smith TK, Kopaniky DR, Oldfield D (1985). Infection about the spine associated with low-velocity missile injury to the abdomen. J Bone Joint Surg Am.

[31] Kumar A, Wood GW, Whittle AP (1998). Low-velocity gunshot injuries of spine with abdominal viscus trauma. J Orthop Trauma.

[32] Maier RV, Carrico CJ, Heimbach DM (1979). Pyogenic osteomyelitis of axial bones following civilian gunshot wounds. Am J Surg.

[33] Venger BH, Simpson RK, Narayaan RK (1989). Neurosurgical intervention in penetrating spinal trauma with associated visceral injury. J Neurosurg.

[34] Ivatury RR, Zubowski R, Psarras P, Nallathambi M, Rohman M, Stahl WM (1988). Intraabdominal abscess after penetrating abdominal trauma. J Trauma.

[35] Kihtir T, Ivatury RR, Simon R, Stahl WM (1991). Management of transperitoneal gunshot wounds of the spine. J Trauma.

[36] Arthurs Z, Kjorstad R, Mullenix P, Rush RM, Sebesta J, Beekley A (2006). The use of damage-control principles for penetrating pelvic battlefield trauma. Am J Surg.

[37] Rhee JM, Martin R (1997). The management of retained bullets in the limbs. Injury.

[38] Long WT, Brien EW, Boucree JB, Filler B, Stark HH, Dorr LD (1995). Management of civilian gunshot injuries to the hip. Orthop Clin North Am.

[39] Petersen W, Beske C, Stein V, Laprell H (2002). Arthroscopical removal of a projectile from the intra-articular cavity of the knee joint. Arch Orthop Trauma Surg.

[40] Lee GH, Virkus WW, Kapotas JS (2008). Arthroscopically assisted minimally invasive intraarticular bullet extraction: technique, indications, and results. J Trauma.

[41] Dillman RO, Crumb CK, Lidsky MJ (1979). Lead poisoning from a gunshot wound. Report of a case and review of the literature. Am J Med.

[42] Volgas DA, Stannard JP, Alonso JE (2005). Current orthopaedic treatment of ballistic injuries. Injury.

[43] Farber JM, Rafii M, Schwartz D (1994). Lead arthropathy and elevated serum levels of lead after a gunshot wound of the shoulder. AJR Am J Roentgenol.

[44] Rehman MA, Umer M, Sepah YJ, Wajid MA (2007). Bullet-induced synovitis as a cause of secondary osteoarthritis of the hip joint: a case report and review of literature. J Med Case Rep.

[45] Schutzer SF, Harris WH (1988). Deep-wound infection after total hip replacement under contemporary aseptic conditions. J Bone Joint Surg Am.

[46] Fender D, Harper WM, Gregg PJ (1999). Outcome of Charnley total hip replacement across a single health region in England: the results at five years from a regional hip register. J Bone Joint Surg (Br).

[47] Blom AW, Taylor AH, Pattison G, Whitehouse S, Bannister GC (2003). Infection after total hip arthroplasty. The Avon experience. J Bone Joint Surg (Br).

[48] Phillips JE, Crane TP, Noy M, Elliott TS, Grimer RJ (2006). The incidence of deep prosthetic infections in a specialist orthopaedic hospital: a 15-year prospective survey. J Bone Joint Surg (Br).

